# Exacerbation of allergic asthma by somatic antigen of *Echinococcus granulosus* in allergic airway inflammation in BALB/c mice

**DOI:** 10.1186/s13071-021-05125-2

**Published:** 2022-01-06

**Authors:** Sara Ghabdian, Sima Parande Shirvan, Mohsen Maleki, Hassan Borji

**Affiliations:** Department of Pathobiology, Faculty of Veterinary Medicine, Ferdowsi University of Mashhad, P.O. Box: 91775-1793, Mashhad, Iran

**Keywords:** Asthma, Somatic products, Helminth therapy, *Echinococcus granulosus*

## Abstract

**Background:**

There is ample evidence demonstrating a reverse relationship between helminth infection and immune-mediated diseases. Accordingly, several studies have shown that *Echinococcus granulosus* infection and hydatid cyst compounds are able to suppress immune responses in allergic airway inflammation. Previous studies have documented the ability of hydatid cysts to suppress aberrant Th2 immune response in a mouse model of allergic asthma. However, there is a paucity of research on the effects of protoscoleces on allergic asthma. Thus, this study was designed to evaluate the effects of somatic antigens of protoscoleces in a murine model of allergic airway inflammation.

**Methods:**

Ovalbumin (OVA)/aluminum hydroxide (alum) was injected intraperitoneally to sensitize BALB/c mice over a period of 0 to 7 days, followed by challenge with 1% OVA. The treatment group received somatic antigens of protoscoleces emulsified with PBS on these days in each sensitization before being challenged with 1% OVA on days 14, 15, and 16. The effects of somatic antigens of protoscoleces on allergic airway inflammation were evaluated by examining histopathological changes, the recruitment of inflammatory cells in the bronchoalveolar lavage, cytokine production in the homogenized lung tissue (IL-4, IL-5, IL-10, IL-17, and IFN-γ), and total antioxidant capacity in serum.

**Results:**

Overall, administration of somatic antigens of protoscoleces exacerbated allergic airway inflammation via increased Th2 cytokine levels in the lung homogenate, recruitment of eosinophils into bronchoalveolar lavage fluid, and pathological changes. In addition, total antioxidant capacity and IFN-γ levels declined following the administration of somatic antigens.

**Conclusions:**

The results revealed that the co-administration of somatic products of protoscoleces with OVA/alum contributed to the exacerbation of allergic airway inflammation in BALB/c mice. Currently, the main cause of allergic-type inflammation exacerbation is unknown, and further research is needed to understand the mechanism of these interactions.

**Graphical Abstract:**

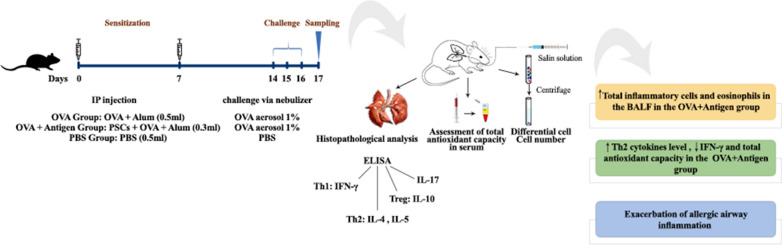

## Background

 Cystic echinococcosis (CE or hydatid disease) is a helminth infection that, despite its high prevalence in tropical regions, has received scant attention [1]. CE is a major endemic health issue with a considerable economic and zoonotic impact worldwide, and is chiefly caused by the larval stage of *Echinococcus granulosus* [2–4]. Adult worms grow in the small intestine of the definitive hosts, which shed egg-laden proglottids in their feces following a prepatent period of about 40–50 days [5, 6]. Unilocular hydatid cysts contain protoscoleces (PSCs) that are mainly found in the liver and lungs of infected intermediate hosts [7].

There is ample evidence supporting the useful roles of helminths and their secretions in the prevention or treatment of allergic and other inflammatory diseases [8, 9]. As shown by in vivo experiments, *E. granulosus* infection dramatically decreases the airway inflammation induced by ovalbumin (OVA) by increasing interleukin 10 (IL-10) and downregulating IL-5 and IL-17A in serum and lung tissues [10]. The effects of the *E. granulosus* laminated layer on nitric oxide (NO) levels was assessed in sera followed by culture with peripheral blood mononuclear cells (PBMC) in patients with severe asthma. The results revealed that laminated layer extracts (LLs) reduced NO production in patients with severe asthma [11]. Similar suppression was obtained by the co-administration of *E. granulosus*-derived cystic fluid that sensitizes the dose of ovalbumin (OVA) in the alum. Immunomodulatory molecules (IMs) in the hydatid cystic fluid have therapeutic potential for suppressing allergic airway inflammation by modifying immune cell activation and cytokine balance through a surge in CD4+CD25+Foxp3+ T cells (Treg cells) [12].

The results confirm the hypothesis that in *E. granulosus* infection, as in some other helminths, hydatid cyst components can also have inhibitory or regulatory effects on allergic airway inflammation. However, few studies have examined the effect of somatic antigens of hydatid cyst PSCs on allergic manifestations in an experimental model of allergic airway inflammation.

The main question is whether PSCs, like other components of the hydatid cyst, can suppress the immune response in allergic airway inflammation. Accordingly, this study aims to investigate the impact of somatic antigens of PSCs on alum-induced allergic immune responses.

## Methods

### Experimental animals

Twenty-four female BALB/c mice (6–8 weeks old) were acquired from Razi Vaccine and Serum Research Institute, Mashhad, Iran. Animals were housed under pathogen-free conditions at the Animal Facility Laboratory of the Faculty of Veterinary Medicine of the Ferdowsi University of Mashhad.

### Parasite

Hydatid cysts were collected from the liver and lungs of naturally infected sheep from the slaughterhouse of Mashhad. For the isolation of PSCs, the cystic fluid was centrifuged at 500×*g* at 4 °C for 5 min, and the isolated PSCs were stored at −20 °C to be used in the next steps.

### Preparation of antigen

The isolated PSCs were sonicated three times (each time for 2 min) on ice using an ultrasonic lab homogenizer (UP 200 W, 26 kHz). The protein concentration of antigens was measured using Bradford assay (Bio-Rad, Hercules, CA, USA). Antigens were stored at −80 °C until use. After the isolation of PSCs, lipopolysaccharide (LPS) was removed from protein antigen.

Triton™ X-114 (CAS 9036-19-5, Sigma-Aldrich, St. Louis, MO, USA) was added to the protein antigen (4 ml in phosphate-buffered saline [PBS]) to a final TX-114 concentration of 1% v/v. The solution was incubated at 4 °C for 30 min with constant stirring. Subsequently, the sample was transferred to a water bath set at 37 °C with constant stirring, and incubated for 10 min followed by centrifugation at 20,000×*g* for 20 min at 37 °C. The upper part containing the protein was separated from the TX-114 layer by means of pipetting. To investigate whether repeated TX-114-assisted extraction increased LPS removal efficiency, the extraction procedure was repeated one, two, and three times [13]. Endotoxin levels in our protein products decreased by as much as 99% from their original content. Upon completion, the upper or watery phase containing the protein was transferred to a new microtube and stored at −20 until use. The lower or colloidal phase containing surfactant and LPS was discarded.

### Murine model of OVA-induced airway inflammation

**Fig. 1:**
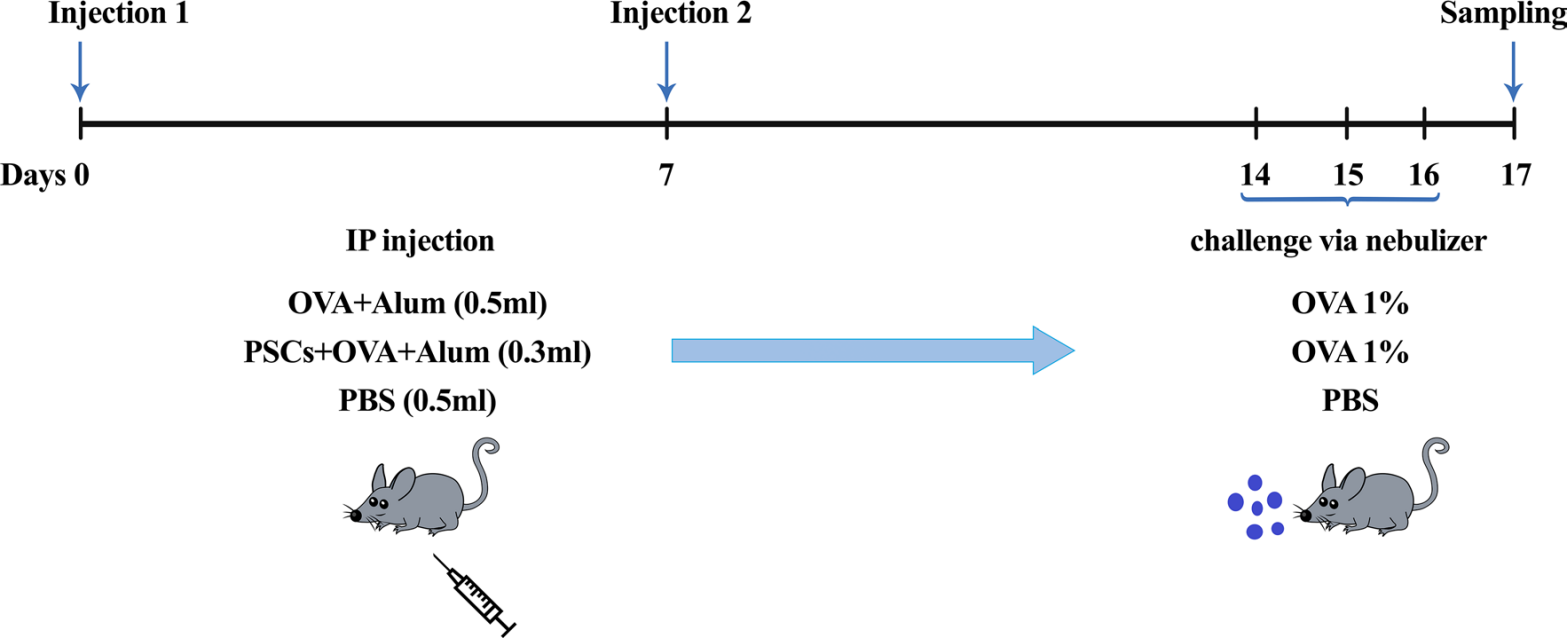
Schedule of an allergic airway inflammation mouse model and treatment protocol. To induce allergic airway inflammation, mice in the OVA and OVA + antigen groups were sensitized on days 0 and 7 with an IP injection of OVA + alum and PBS. The OVA + antigen group received PSCs emulsified with PBS on these days. The PBS group received 0.5 ml of PBS on days 0 and 7. After the second sensitization, mice were challenged by exposure to aerosols of OVA and PBS on days 14, 15, and 16. Collection of BALF, lungs, and blood serum was performed 24 h after the last exposure. *PSCs* protoscoleces, *OVA* ovalbumin, *Alum* aluminum hydroxide, *IP* intraperitoneal, *PBS* phosphate-buffered saline, *BALF* bronchoalveolar lavage fluid

To evaluate the effects of PSCs of the hydatid cyst in the acute model of allergic airway inflammation, the mice were divided into three groups, each containing eight mice, as follows:

(i) The negative control group (PBS group) received intraperitoneal (IP) injections of PBS only on days 0 and 7 and challenges with PBS aerosol via nebulizer (UltraNeb™ 2000; DeVilbiss, Mannheim, Germany) on days 14, 15, and 16.
(ii) The positive control group (OVA group) was sensitized with two IP injections of OVA (Sigma-Aldrich, St. Louis, MO, USA) precipitated with aluminum hydroxide (alum, Sigma-Aldrich) on days 0 and 7 and challenged with OVA aerosol by a nebulizer on days 14, 15, and 16.
(iii) The treatment group (OVA + antigen group) was sensitized with two IP injections of OVA + alum on days 0 and 7. This group received 20 μg somatic extract on these days and challenge with OVA aerosol on days 14, 15, and 16 (Fig. 1).

### Total and differential cell counts of bronchoalveolar lavage

Twenty-four hours after the final OVA challenge, mice were anesthetized and their trachea was exposed and cut just below the larynx to obtain bronchoalveolar lavage fluid (BALF). Bronchoalveolar cells were prepared by lung lavage after three injections of 0.4 ml cold PBS into the lungs. A total of 1 ml BALF was collected and centrifuged at 4000 rpm at 4 °C for 5 min. The bottom fluid was suspended in 100 μl of cold PBS, and the total number of inflammatory cells infiltrating the BALF was determined by a hemocytometer. For a differential cell count, a smear of the cell pellet of BALF was prepared and stained with Giemsa.

### Assessment of the total antioxidant capacity in serum

Blood samples were taken from the animal’s heart using an insulin syringe and then transferred to sterile microtubes. After storage at room temperature, the samples were centrifuged at 8000 rpm for 10 min and the serum was separated. Afterward, they were transferred to new microtubes and stored at −20 °C for further analysis. The total antioxidant power in the serum was estimated by measuring the ferric-reducing ability using the Naxifer™ Total Antioxidant Capacity Assay Kit (TAC). The absorbance was measured at 490 nm using a UV–visible spectrophotometer.

### Histopathological analysis of lung tissues

The lungs were dissected from the chest cavity as follows. The left lung of each mouse was removed and used for the preparation of pathological sections. The tissue was subsequently fixed in a 10% neutral-buffered formalin solution. After 24 h fixation with formalin, 3–5-micron sections were obtained using a microtome and stained with hematoxylin–eosin (H&E) to assess the extent to which inflammatory cells infiltrate a periodic acid–Schiff (PAS) stain for the hyperplasia and metaplasia of goblet cells. After staining, the stained sections were microscopically evaluated, and pathological changes were scored on a scale of 0–4. The degree of inflammatory cell infiltration was subjectively classified as absent, minimal, slight, moderate, or significant. In addition, structural changes such as goblet cell metaplasia were evaluated to determine the presence of inflammatory cells.

### Evaluation of cytokines in the homogenized lung tissues

The right lung was frozen in liquid nitrogen and stored at −80 °C for cytokine detection. On the day of analysis, frozen lungs were thawed and weighed, and 100 mg of wet tissue was homogenized with a tissue homogenizer in 1 ml homogenization buffer containing KCL (0.5 M), Tris–Cl (1 M, pH 9), and Triton X-100. The homogenates were centrifuged at 10,000×*g* for 10 min at 4 °C, and the supernatants were used for cytokine detection using an enzyme-linked immunosorbent assay (ELISA). The cytokine levels including IL-4, IL-5, IL-10, IL-17, and interferon gamma (IFN-γ) were measured in lung tissue homogenates using a sandwich ELISA (R&D Systems, Minneapolis, MN, USA) according to the manufacturer’s instructions.

### Statistical analysis

All data were analyzed using GraphPad Prism software (version 8). Statistical significance of differences between groups was determined using one-way analysis of variance (ANOVA) and Kruskal–Wallis tests. *P*-values < 0.05 were considered significant.

## Results

### Total cell count in BALF

**Fig. 2:**
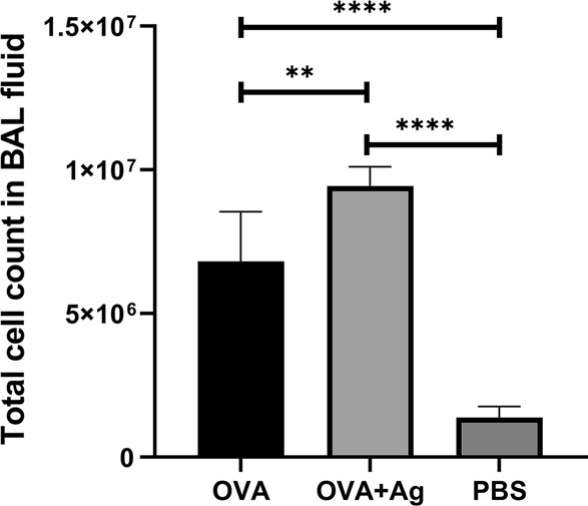
Effect of hydatid cyst PSCs on total inflammatory cells and eosinophils in BALF of BALB/c mice sensitized and challenged with OVA compared with OVA and PBS groups. Error bars show standard deviation (SD). *P* < 0.05; one-way ANOVA test

The number of inflammatory cells was counted 24 h after the last OVA aerosol challenge, as shown in Fig. 2. The results of differential cell counting of cells in the BALF showed that mice receiving OVA + antigen exhibited a dramatic increase in the total cell count in the BALF, and the predominant cells were eosinophils. A remarkable growth in the number of eosinophils and inflammatory cells of the BALF was observed in the OVA + antigen group following administration of PSCs twice during sensitization with OVA in comparison with the OVA group (ANOVA: *F*_(2, 12)_ = 71.01, *P* < 0.0001). No infiltration of inflammatory cells was observed in the PBS group of mice sensitized and challenged with PBS.

### Cytokine analysis in lung homogenate

**Fig. 3:**
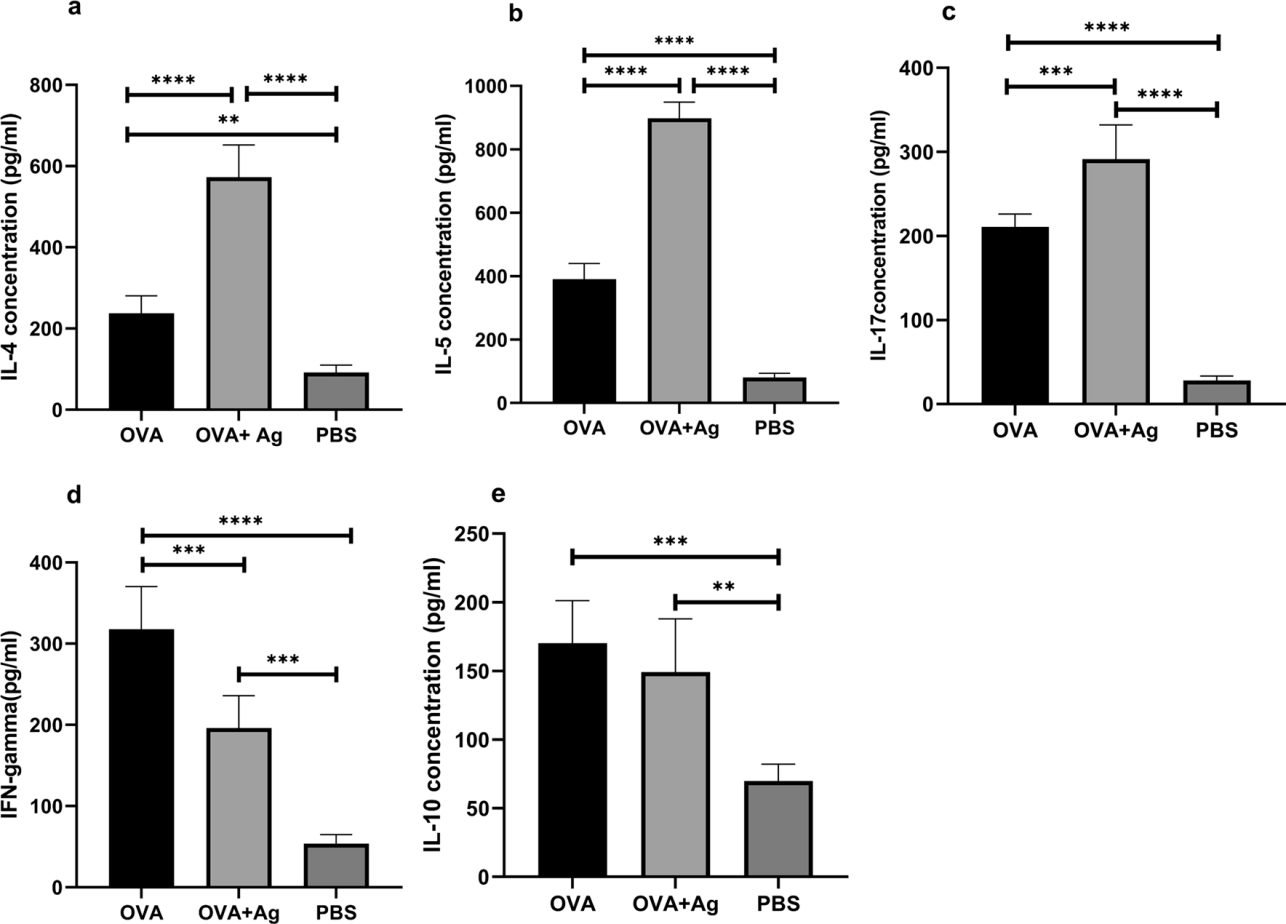
Effect of hydatid cyst PSCs on the levels of cytokines in the lung tissue homogenates. Levels of these cytokines were quantified by specific ELISA assay kits. Cytokine responses (**a** IL-4, **b** IL-5, **c** IL-17, **d** IFN-γ, **e** IL-10) were measured in mice given OVA/alum in the presence or absence of PSCs. Error bars indicate standard deviation (SD). *P* < 0.05; one-way ANOVA. *ELISA* enzyme-linked immunosorbent assay

The production of cytokines including IL-4, IL-5, IL-17, IL-10, and IFN-γ was measured in the lung homogenate using the sandwich ELISA (Fig. 3). Our research revealed higher levels of Th2 cytokines including IL-4 and IL-5 following PSC administration in comparison with the OVA group (ANOVA: *F*_(2, 12)_ = 108.3, *P* < 0.0001 and ANOVA: *F*_(2, 12)_ = 489.0, *P* < 0.0001, respectively) (Fig. 3 a, b). The IL-17 measurement indicated that the level of IL-17 was significantly higher in the OVA + antigen group than in the OVA group (ANOVA: *F*_(2, 12)_ = 142.7, *P* < 0.0001) (Fig. 3c). Following administration of PSCs, Th1 cytokines including IFN-γ levels decreased significantly in the OVA + antigen group compared with the OVA group. (ANOVA: *F*_(2, 12)_ = 58.56, *P* < 0.0001) (Fig. 3d).

Additionally, no significant difference in the level of IL-10 was observed between the mice receiving PSCs and those in the OVA group (ANOVA: *F*_(2, 12)_ = 15.99, *P* = 0.0004) (Fig. 3e).

### Histopathology of the lungs

**Fig. 4:**
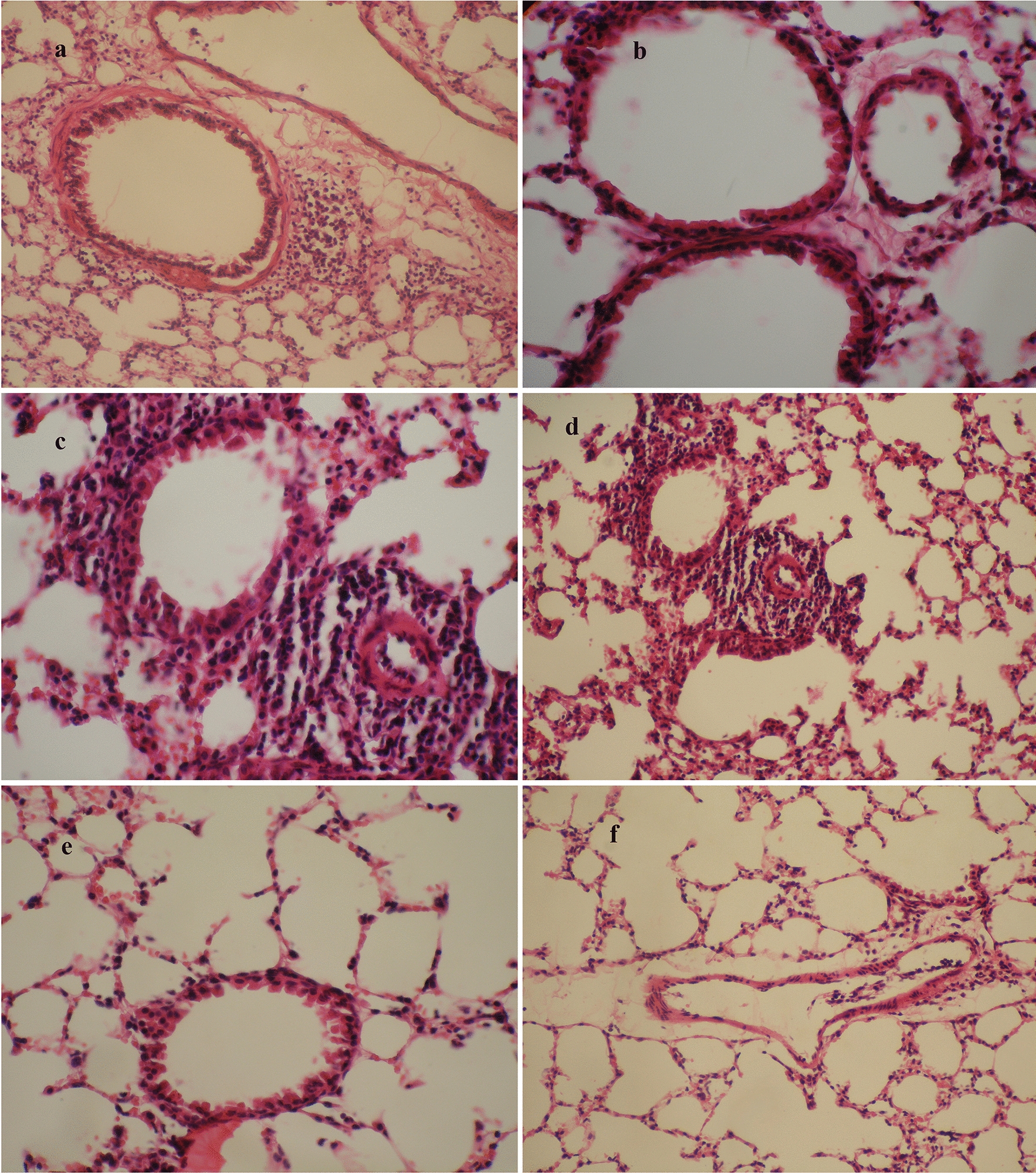
Histopathological sections of lung tissues from BALB/c mice in the OVA group (**a** ×20, **b** ×40), OVA + antigen group (**c** ×40, **d** ×20), and PBS group (**e** ×20, **f** ×20) stained with H&E. *H&E* hematoxylin–eosin staining

The histopathological changes were greater in OVA-induced mice after PSC administration than in other groups. Histopathological findings showed marked infiltration of inflammatory cells, predominantly mononuclear cells such as eosinophils, in perivascular and peribronchial areas of the lungs in the OVA + antigen group compared with the OVA and PBS groups (Kruskal–Wallis: *χ*^2^ = 12.519, *df* = 2, *P* = 0.002) (Fig. 4).

**Fig. 5:**
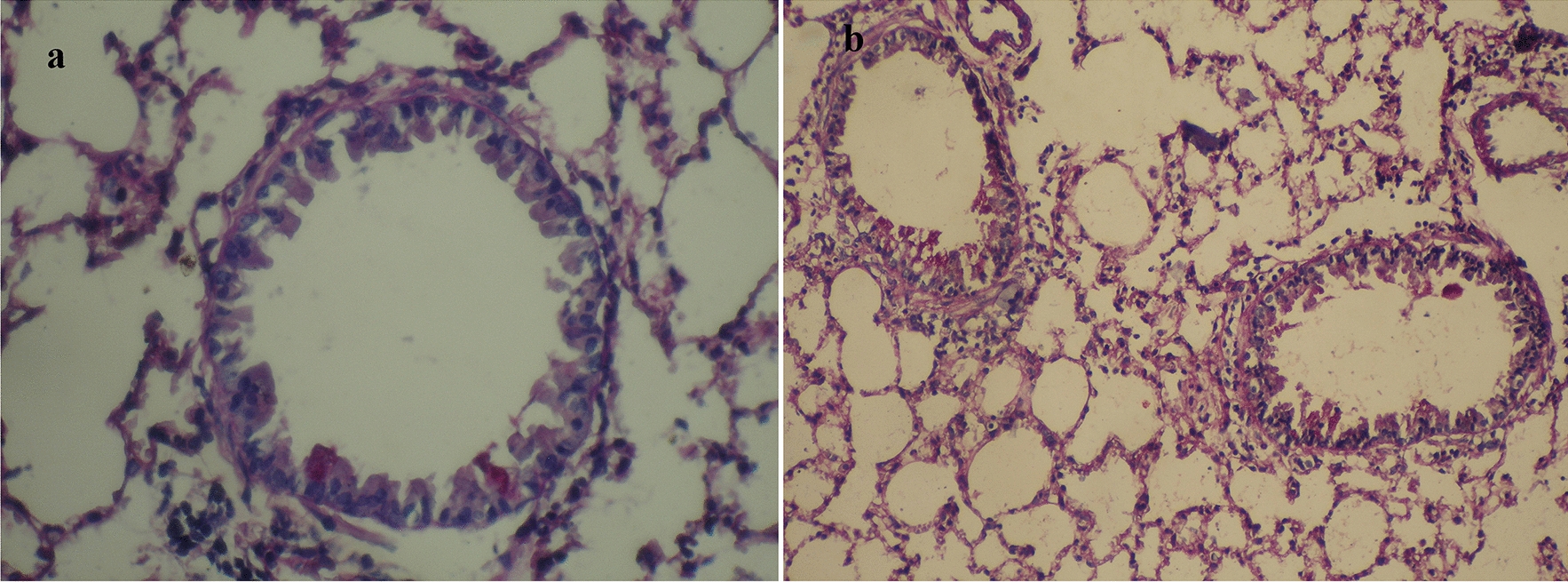
Effect of hydatid cyst PSCs on development of OVA-induced airway inflammation in a mouse model stained with PAS (**a** ×40, **b** ×20). *PAS* periodic acid–Schiff staining

Histopathological results by PAS staining in the OVA + antigen group suggested structural changes in airways such as marked goblet cell metaplasia and elevated mucus production compared with the OVA and PBS groups. (Kruskal–Wallis: *χ*^2^ = 12.715, *df* = 2, *P* = 0.002) (Fig. 5). Also, no pathological changes were observed in the mice sensitized and challenged with PBS.

### Evaluation of total antioxidant capacity by ferric-reducing antioxidant power of plasma (FRAP) assay

**Fig. 6:**
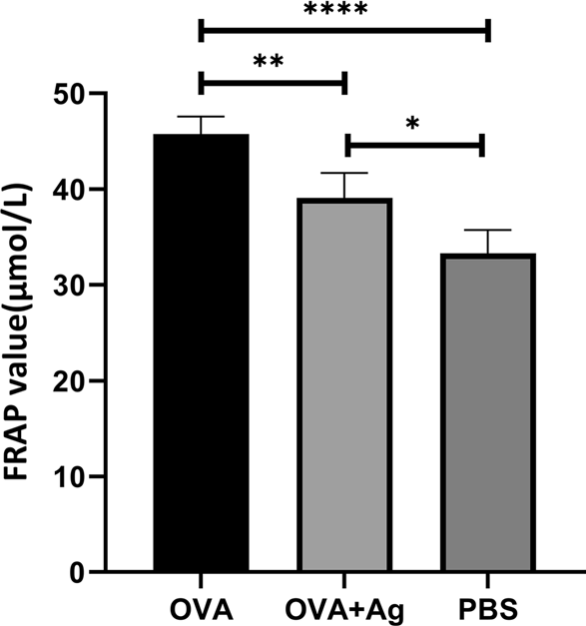
Effect of hydatid cyst PSCs on the ferric-reducing antioxidant power in serum expressed as μmol/l. Error bars show standard deviation (SD). *P* < 0.05; one-way ANOVA test

Antioxidant capacity was evaluated in blood serum. It was found that the total antioxidant potential in serum, as measured by FRAP, decreased markedly in the OVA + antigen group compared with the OVA and PBS groups (ANOVA: *F*_(2, 10)_ = 28.99, *P* < 0.0001) (Fig. 6).

## Discussion

Recent years have witnessed major advances in helminth therapy, which has led to the introduction of new strategies for the treatment of immune-mediated diseases. Most studies suggest that helminth infections can provoke symptoms of allergy and worm infestation by establishing a chronic infection, leading to reduced eosinophilia and allergic inflammation in the airways [9]. To the best of our knowledge, this is the first report about the effect of somatic antigens of PSCs on allergic airway inflammation in a murine model. There is ample experimental evidence that the co-administration of somatic products of PSCs with OVA/alum despite the presence of laminated layer, *E. granulosus* antigen B (Eg AgB), and hydatid fluid can exacerbate allergic asthma [10–12, 14]. Moreover, some intestinal helminths such as *Toxocara* spp., schistosomes, and *Toxoplasma gondii* are parasites inversely associated with allergic disorders [15].

In contrast, several experimental and clinical studies have reported inconsistent findings. Researchers reported a direct association between helminth infections and helminth-derived molecules in asthma and other atopic diseases. Some reports suggest that intestinal helminths such as *Ascaris lumbricoides*, *Strongyloides stercoralis*, *Toxocara cati*, and *Anisakis* species can be directly linked to allergic-type inflammation [8].

In 2015, Ahumada et al. revealed that enhanced Th2-biased immune response along with IgE responses to *Ascaris* extract act as a risk factor for the pathogenesis of asthma [16]. In 2020, Bakhshani et al. showed that oral administration of embryonated *T. cati* eggs after sensitization and OVA challenge exacerbated airway hyperresponsiveness, eosinophilia, and pulmonary inflammation while raising the IL-5 level in the lungs of mice infected with *T. cati* [17].

In our study, the administration of PSCs induced drastic histopathological changes, leading to extensive penetration of inflammatory cells into the BALF, elevated levels of IL-4, IL-5, and IL-17 in the lung homogenate, and reduced total antioxidant capacity in serum in the OVA + antigen group in comparison with the OVA and PBS groups.

Different effects can be attributed to the high allergenicity of some helminths and also species-specific disparities in the immune polarization, although the exact cause of this mechanism is unclear [18].

It is hypothesized that in the absence of helminth infection, allergenic proteins can influence the host immune response. According to previous studies, specific antigens from *E. granulosus* are capable of promoting a Th2 cytokine profile in order to induce IgE secretion, acting as the origin of allergic reactions during echinococcosis [19]. The findings of this study demonstrate that IgE can contribute to host protection against parasitic agents, but this protective function was never documented in larval cestodes [20].

In particular, antigen B (AgB), antigen 5 (Ag5), cyclophilin (EA21), heat shock protein 70 (Hsp70), and elongation factor 1-beta/delta (EF-1 β/δ) have been identified as allergenic molecules in fluid cysts and PSCs. People with CE have IgE to parasite antigens AgB, a protease inhibitor, Ag5, a serine protease, and EA21 [21]. In patients with progressive CE disease, AgB can produce IL-4 and IL-13, provoking immunopathology-associated Th2 polarization [22]. It has been shown that AgB is able to induce an anti-inflammatory phenotype in macrophages, which is useful for the suppression of allergic airway eosinophilic inflammation [14]. There are no details about the molecular mechanisms involved in the immunogenic and allergic features of AgB.

Ortona et al. in 2002 demonstrated that *E. granulosus* cyclophilin (specific antigens that exist in PSCs and fluids) plays a role in allergic symptoms associated with CE, which can compromise or protect the host [19].

In most patients with CE, Eg EF-1 β/δ can tilt Th1/Th2 cytokine activation towards Th2 polarization in a preferential manner [23]. However, most proteins are not allergens, and the reason that just a minority of antigens possess allergenic properties is not clear [21].

Classically, asthma is treated as a Th2 disease related to enhanced IgE and eosinophilic inflammation, which is crucial to asthma pathophysiology in the airway [24]. IL-4, as a central mediator of asthma, plays a pivotal role in the switching of the IgE isotype in B cells. IL-5 promotes the proliferation and differentiation of eosinophils [25]. In this study, an enhanced Th2-biased immune response (IL-4, IL-5) was observed following administration of PSCs in OVA-induced mice relative to the OVA and PBS groups. These results suggest that the activation of type-2 immunity may also affect the reactivity of airways and the number of eosinophils in BALF.

Consistent with the high level of Th2 cytokines, a high concentration of IL-17 was detected in the OVA + antigen group lung homogenate compared with the OVA and PBS groups. This suggests that somatic antigens of PSCs may increase the level of Th17. Given the simultaneous surge in both Th2 and IL-17 in the OVA + antigen group, IL-17 co-transferred with Th2 cytokines can promote eosinophilic airway inflammation mediated by Th2 cells by improving the expression of eotaxin and eotaxin-1 as major chemokines for recruitment of eosinophils into the airways [26–28]. These findings show that IL-17 is involved in the development of allergic asthma with Th2 cytokines.

Other cell populations including Treg cells that are essential for the prevention of lung inflammation act as immunoregulatory factors [29]. Following the measurement of its concentration in the lung homogenate, the levels of IL-10 in the OVA + antigen group were lower than those in the OVA group, but this difference was nonsignificant.

The level of type-1-specific cytokines such as IFN-γ (a representative cytokine derived from Th1 cells) increases with the exacerbation of asthma. IFN-γ prevents Th2 cell-mediated eosinophilic inflammation, airway hyperresponsiveness (AHR), and mucus production [16].

Following the surge in Th2 cytokines, a significant reduction was observed in IFN-γ levels in the lungs of mice in the OVA + antigen group compared with the OVA and PBS groups.

The FRAP assay was used to determine the total antioxidant power at 490 nm. Nadeem et al. in 2005 showed that in patients with acute exacerbations, the total antioxidant capacity of plasma decreased. Severe exacerbation of asthma was linked to enhanced oxidative stress. Also, reduced antioxidant power of plasma was positively correlated with inflammation, acting as a causative factor in the pathogenesis of asthma [30]. The valuation of total antioxidant capacity in blood serum following PSC administration indicated a remarkable reduction in the OVA + antigen group in comparison with the OVA and PBS groups. Also, previous research has underlined the importance of oxidant–antioxidant balance for natural pulmonary function [31]. However, the inflammatory response in asthma may be rooted in multiple pathways [11].

Histopathological findings revealed that the co-administration of somatic antigens of PSCs with OVA intensified airway inflammation. Also, abundant bronchiolar goblet cells appeared in the bronchiolar epithelium in the OVA + antigen group in comparison with the OVA and PBS groups. Hyperplasia in bronchioles and bronchi and mucus hypersecretion in bronchi were detected in the OVA + antigen group by PAS staining. Given that IL-13 is linked to goblet cell hyperplasia and mucus production in airway epithelial cells [32], and represents a critical pathological feature of the allergic response [24], its administration during OVA sensitization may raise IL-13 levels. However, IL-13 cytokine levels were not measured in the lung homogenate in the present study. Thus, it is impossible to draw any conclusions about intergroup differences in terms of lung homogenate cytokine levels.

## Conclusions

The results suggest that the co-administration of somatic products of PSCs with OVA/alum exacerbated allergic airway inflammation in BALB/c mice. At present, the underlying cause of exacerbated allergic-type inflammation is unknown, and further studies are needed to shed light on the mechanism of these interactions. Therefore, further research is required to determine whether allergenic molecules are involved in allergic symptoms. Another important challenge that should be highlighted is PSC-specific allergens that can exacerbate inflammation. Future studies need to identify the mechanisms that control the aggravating effects of helminth and helminth-derived molecules on allergic-type inflammation.

## Data Availability

All data generated or analyzed during this study are included in this published article and its additional file.

## Abbreviations

PSCs: Protoscoleces
LL: Laminated layer extract
LPS: Lipopolysaccharide
PBS: Phosphate-buffered saline
OVA: Ovalbumin
Alum: Aluminum hydroxide
IP injection: Intraperitoneal injection
BALF: Bronchoalveolar lavage fluid
H&E: Hematoxylin–eosin
PAS: Periodic acid–Schiff
ELISA: Enzyme-linked immunosorbent assay
FRAP: Ferric-reducing antioxidant power of plasma
Eg AgB: *Echinococcus granulosus* antigen B
AHR: Airway hyperresponsiveness
